# p21-Activated Kinase 3 (PAK3) Is an AP-1 Regulated Gene Contributing to Actin Organisation and Migration of Transformed Fibroblasts

**DOI:** 10.1371/journal.pone.0066892

**Published:** 2013-06-20

**Authors:** Nina Holderness Parker, Howard Donninger, Michael J. Birrer, Virna D. Leaner

**Affiliations:** 1 Division of Medical Biochemistry, Faculty of Health Sciences, University of Cape Town, Institute of Infectious Disease and Molecular Medicine, Cape Town, South Africa; 2 Department of Medicine, James Graham Brown Cancer Center, Molecular Targets Program, University of Louisville, Louisville, Kentucky, United States of America; 3 Harvard Medical School, Gynecologic Cancer Research Program, Gillette Center for Gynecologic Oncology, Massachusetts General Hospital, Boston, Massachusetts, United States of America; Beatson Institute for Cancer Research Glasgow, United Kingdom

## Abstract

Activating Protein 1 (AP-1) plays a vital role in cell proliferation, differentiation and apoptosis. While de-regulation of AP-1 has been linked to many cancers, little is known regarding its downstream transcriptional targets that associate with cellular transformation. Previous studies identified PAK3, a serine/threonine kinase, as a potential AP-1 target gene. PAK3 has been implicated in a variety of pathological disorders and over-expression of other PAK-family members has been linked to cancer. In this study, we investigate AP-1 regulation of PAK3 expression and the role of PAK3 in cJun/AP-1-associated cellular transformation. Our results showed elevated PAK3 expression at both the mRNA and protein level in cJun-over-expressing Rat1a fibroblasts, as well as in transformed human fibroblasts. Elevated PAK3 expression in cJun/AP-1 over-expressing cells associated with a significant increase in PAK3 promoter activation. This increased promoter activity was lost when a single putative Jun binding site, which can bind AP-1 directly both *in vitro* and *in vivo,* was mutated. Further, inhibition of PAK3 using siRNA showed a regression in the cell morphology, migratory potential and actin organisation associated with AP-1 transformed cells. Our study is a first to describe a role for AP-1 in regulating PAK3 expression and suggest that PAK3 is an AP-1 target required for actin organization and migration observed in transformed cells.

## Introduction

Activating Protein 1 (AP-1) regulates the expression of genes involved in cell proliferation, differentiation and apoptosis, regulating the cellular physiology in response to many stresses [1]; [2]. Constitutive over-expression of cJun/AP-1 has been shown to cause cellular transformation within Rat1a cells [3] and this transformed phenotype includes changes in cell proliferation, morphology and the induction of anchorage-independent growth [1]. The activation of AP-1 has been linked to deregulated cell proliferation, angiogenesis, invasion, metastasis and tumour progression [4], and its over-expression has been observed in breast, ovarian, endometrial, colon, cervical and lung cancers [5]–[10]. Although there is substantial evidence for AP-1′s role within oncogenesis, very little is known about which of its target genes are essential for these processes [11]. Due to the diverse role that AP-1 plays within the cell, it is thought there is a subset of its target genes whose deregulation is vital for the maintenance of the transformed phenotype [12]. Thus far, a limited number of genes have been identified to play a partial role in AP-1 associated transformation; including a hairpin-binding epidermal growth factor (HB-EGF) [13], a scaffold protein, SSeCKS [12]; [14] and an extra cellular matrix protein, SPARC [14]; [15]. A previous study using differential display and microarray analysis identified p21-Activated Kinase 3 (PAK3), a member of the PAK-family of serine/threonine kinases, to be significantly up-regulated in AP-1 over-expressing cells [1]; [16].

The PAK family of proteins is divided into two subsets, Group 1 (PAK1, PAK2 and PAK3) and Group 2 (PAK4, PAK5 and PAK6) [17]. Group 1 proteins are reported to be the primary effectors of the small GTPases and are activated when bound by the activated form (GTP-bound) of CDC42 and Rac1 [18]. When activated, Group I PAK proteins have been shown to play a role in the regulation of survival, gene transcription, signal transduction, cytoskeletal reorganization, cell morphology and motility [17]. Originally PAK3 was thought to be predominantly expressed in the brain, with its major function in the embryonic development of the brain and neurons [17]. Thus, it is not surprising that there is a strong link between PAK3 and pathological disorders such as certain X-linked mental retardation syndromes [19] and learning problems associated with synaptic plasticity [20]. However, over-expression of PAK3 has been shown in thymic carcinoids, suggesting a possible link between PAK3 and oncogenesis [21]. While there is limited reports showing PAK3 expression in cancers, PAK1 over-expression has been linked to ovarian, breast, bladder and lymph cancers, while elevated PAK2, PAK4 and PAK6 expression has also been observed in prostate cancer [22]. PAK4 is also reported to be elevated in 78% of cancer cell lines and plays a role in focus formation [23]. PAK1 and PAK3 have also been shown to stimulate cell migration and, through the phosphorylation of Raf1, cause anchorage-independent growth [24]. There is thus evidence that over-expression of the PAK proteins associate with cancer development, however, little is known regarding the factors that regulate their expression. The aim of this study was to investigate the regulation of PAK3 expression by AP-1 and determine the effect of inhibiting PAK3 expression on cancer cell biology associated with AP-1 transformation. We report that cJun/AP-1 regulates elevated PAK3 expression by directly binding to the PAK3 promoter, and PAK3 expression is required for cell migration and actin organization in transformed fibroblasts.

## Results

### Over-expression of cJun/AP-1 Results in Increased PAK3 Expression

**Figure 1:**
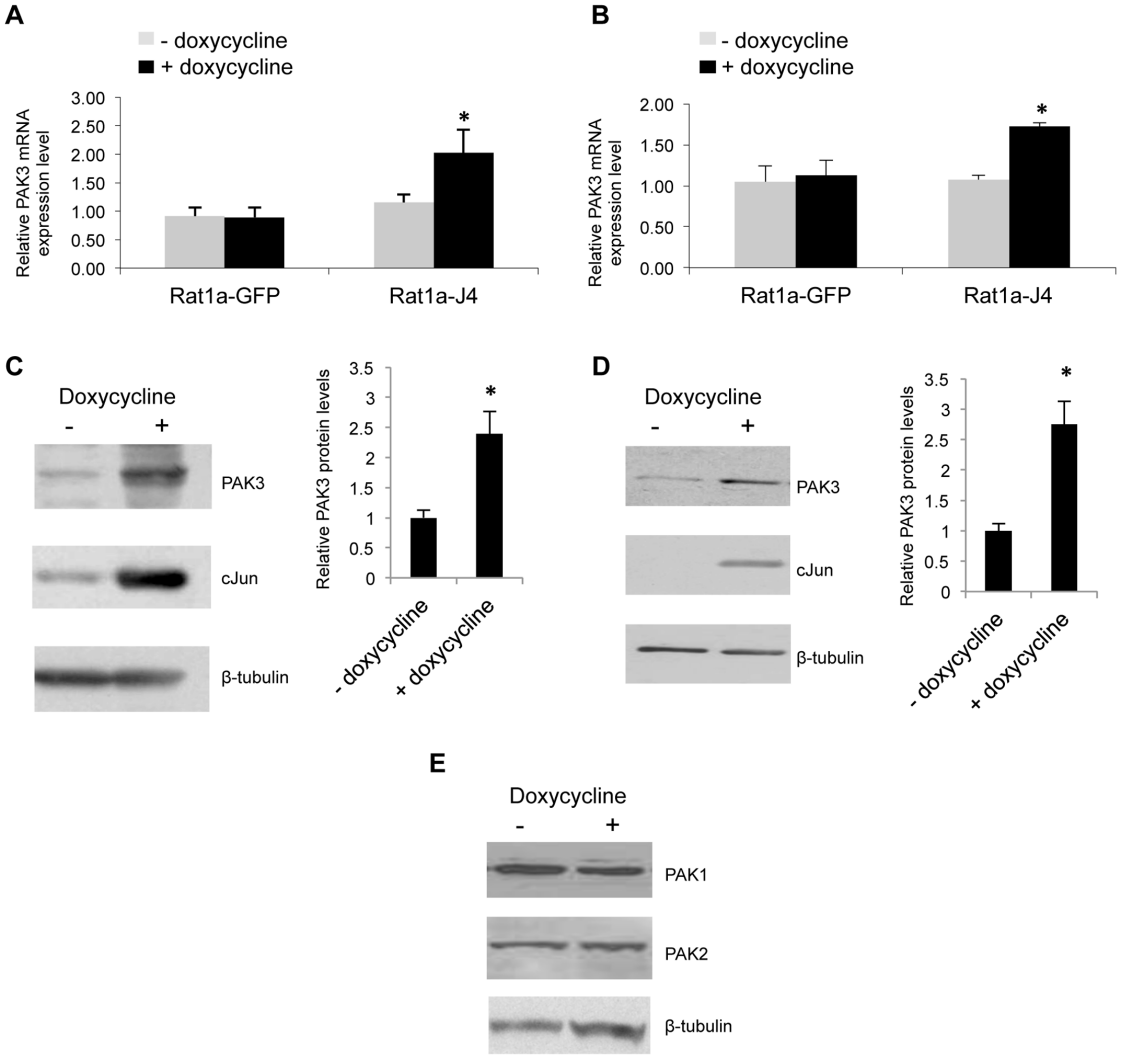
Over-expression of cJun/AP-1 results in increased PAK3 expression. **A & B:** Real time RT-PCR analyses of PAK3 mRNA expression in Rat1a-GFP control cells and Rat1a-J4 cells grown in the absence and presence of doxycycline. Cells were grown in anchorage-dependant (**A**) and anchorage-independent (**B**) conditions. Results are the mean±S.E. of at least three independent experiments. (*p≤0.05) **C & D:** Western blot analysis showing PAK3 and cJun expression levels in anchorage-dependent (**C**) and anchorage-independent (**D**) growth conditions in Rat1a-J4 cells grown with or without doxycycline. Bar graphs show PAK3 protein levels, relative to the β-tubulin levels for each sample,±S.E.M over three independent experiments. (*p≤0.05) **E:** Western blot analysis showing PAK1 and PAK2 protein levels in response to doxycycline induction in anchorage-independently grown inducible Rat1a cells.

Expression analysis identified PAK3 mRNA, amongst others targets, to be up-regulated in cJun/AP-1 over-expressing cells [16]. To independently confirm PAK3 expression in response to cJun/AP-1 induction, qRT-PCR and Western blot analysis was performed using control rat fibroblasts, Rat1a-GFP, and a rat fibroblast cell line with doxycycline-inducible cJun expression, Rat1-J4. Cells were grown in anchorage-dependent (Fig. 1A & C) and anchorage-independent (Fig. 1B, D & E) conditions and PAK3 mRNA and protein expression levels assayed. Our results show that PAK3 mRNA (Fig. 1A & B) and protein levels (Fig. 1C & D) were found to be significantly elevated in response to induced cJun/AP-1 expression in both growth conditions. The expression of other Group 1 PAK proteins, PAK1 and PAK2, remained relatively unchanged in response to the cJun/AP-1 induction (Fig. 1E). Thus, the elevated PAK3 expression in response to cJun/AP-1 induction suggests that PAK3 is an AP-1 responsive gene.

### cJun/AP-1 Activates PAK3 Promoter Activity

**Figure 2:**
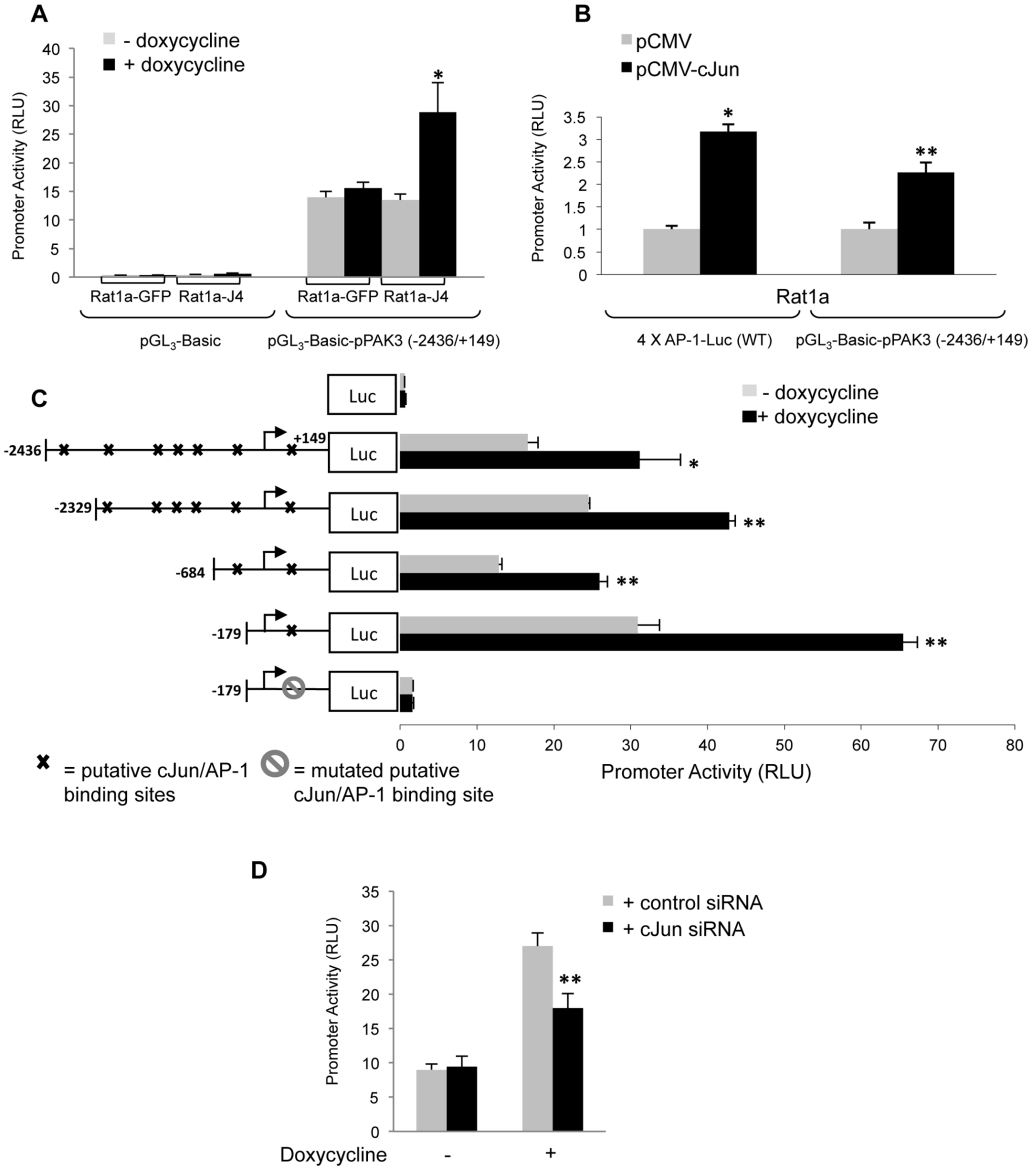
cJun/AP-1 over-expression causes increased PAK3 promoter activity via a binding site at position (+52/+60). The (−2436/+149) region of the PAK3 promoter, containing several putative cJun/AP- binding sites, was cloned into the pGL_3_-Basic reporter vector. **A:** Luciferase promoter reporter assays performed in control cells, Rat1a-GFP, and Rat1a-J4 cells transiently transfected with empty vector, pGL_3_-Basic, and PAK3 promoter construct containing vector, pGL_3_-Basic-pPAK3 (−2436/+149). Cells were grown in the absence and presence of doxycycline. **B:** Luciferase promoter reporter assays performed in the parental rat fibroblast cell line, Rat1a, transfected with either pCMV-cJun or empty pCMV vector with the PAK3 promoter construct containing vector, pGL_3_-Basic-pPAK3 (−2436/+149). A plasmid containing four AP-1 binding sites, 4 X AP-1-Luc, was used as a control showing cJun activation. **C:** Luciferase reporter promoter assays for deletion constructs of the PAK3 promoter region (−2436/+149), (−2329/+149), (−684/+149) and (−179/+149) as well as a (−179/+149) with a mutated cJun/AP-1 binding site at position (+52/+60), in the presence and absence of doxycycline-induced cJun/AP-1 expression. **D:** Luciferase reporter assays, using the (−179/+149) PAK3 promoter construct, showing the effect of transient cJun inhibition on PAK3 promoter activity in the absence and presence of doxycycline induced cJun/AP-1 over-expression. Results show the mean±S.E. of experiments performed in triplicate and repeated at least three times. (*p≤0.05 and **p≤0.01).

To investigate AP-1 regulation of PAK3 gene expression, the (−2436/+149) rat PAK3 promoter region was cloned into the luciferase reporter vector, pGL_3_-Basic. Transient transfection of the (−2436/+149) rat PAK3 promoter plasmid, pGL_3_-Basic-pPAK3 (−2436/+149), into Rat1a-J4 cells showed a significant increase in promoter activity in doxycycline-treated cells compared to controls (Fig. 2A). This effect was not observed in Rat1a-GFP control cells. As an alternative method of AP-1 over-expression, transient transfection of pCMV-cJun into Rat1a parental cells showed a significant, 2.3 fold increase in the (−2436/+149) PAK3 promoter activity (Fig. 2B). A reporter construct containing four AP-1 binding sites, 4 X AP-1-Luc (WT), was used as a positive control of AP-1 activity in pCMV-cJun transfected cells. In order to identify regions within the (−2436/+149) PAK3 promoter that may be responsive to AP-1, a bioinformatic analysis of this region, using MatInspector [25] and ConReal [26], was done to identify potential cJun binding sites. Several putative cJun/AP-1 TRE elements were identified within the (−2436/+149) region of the PAK3 promoter (Fig. 2C). Deletion constructs of the PAK3 promoter, eliminating various putative cJun binding sites, all showed increased promoter activity in response to doxycycline-induced cJun/AP-1 expression. Interestingly, the (−179/+149) PAK3 promoter construct, despite containing only a single putative AP-1 binding site at position (+52/+60) with the sequence of TGACGTCA, retained the significant response to cJun/AP-1 seen in all the PAK3 promoter constructs. This AP-1 binding site and the region surrounding it was found to be conserved in other species, such as human, mouse and dog, while the site itself had one nucleotide change but retains the features of an AP-1 binding site. Site-directed mutagenesis of this putative AP-1 binding site at (+52/+60) to ACGCGTTT (where the underlined regions highlight the mutated bases) of the (−179/+149) promoter construct resulted in a significant loss in PAK3 promoter activity. This shows that the AP-1 binding site at position (+52/+60) is necessary for the expression of the PAK3 gene in response to the doxycycline induction of cJun/AP-1. Furthermore, to ascertain whether this increased promoter activity, via the (+52/+60) site, seen in response to doxycycline treatment, is directly through the presence of increased cJun/AP-1, siRNA was used to transiently knock-down cJun protein levels. This was done with the wildtype (+179/+149) promoter construct in the control and doxycycline-treated cells. Inhibition of cJun did not affect the basal activity of the uninduced (+179/+149) promoter construct, while the knock-down of cJun affected the increased promoter activity seen in doxycycline-induced cJun over-expressing cells (Fig. 2D). This result provides further evidence that cJun is required for PAK3 promoter activation.

### AP-1 Binds Directly to the (+52/+60) PAK3 Promoter Region

**Figure 3:**
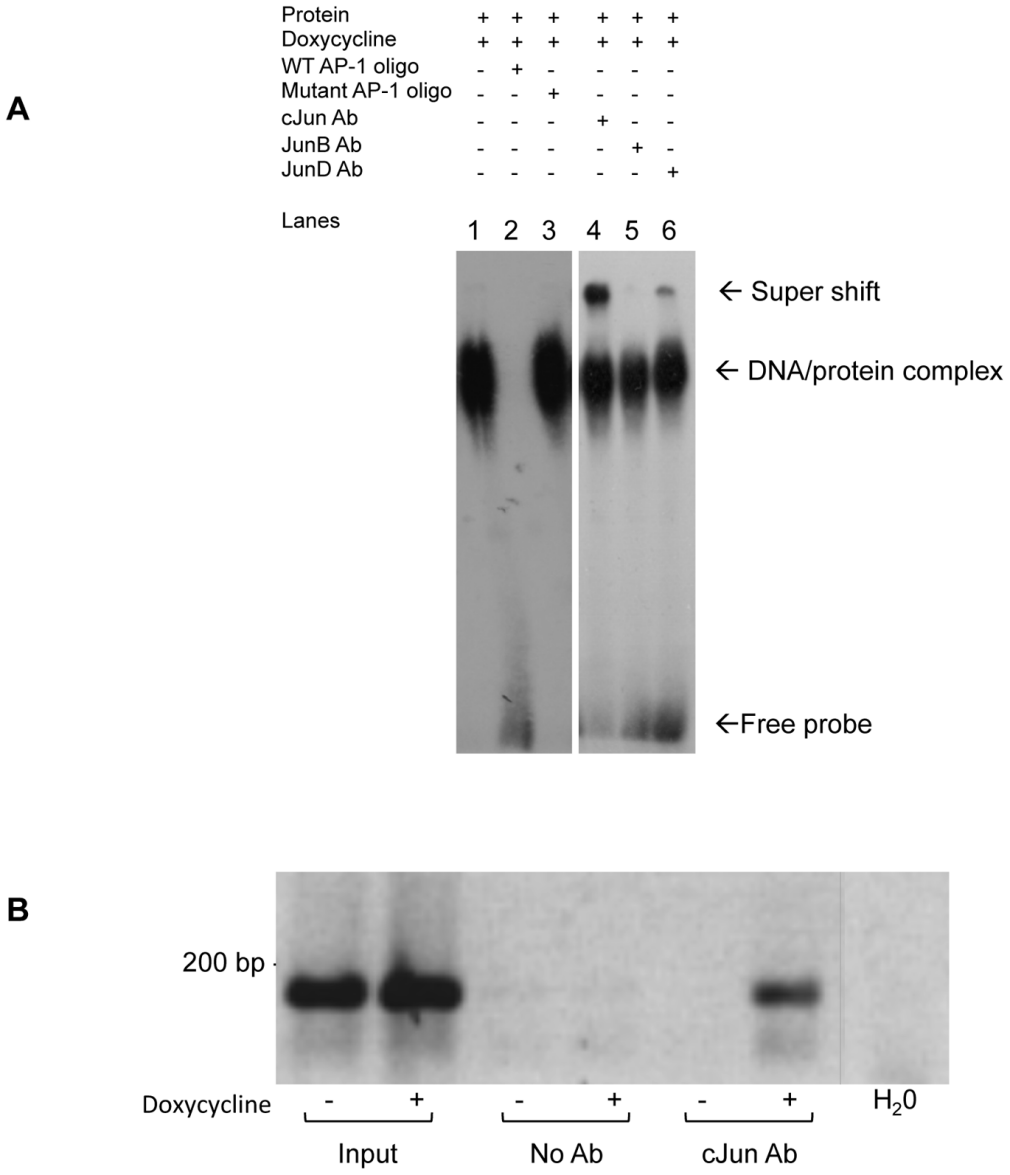
AP-1 binds directly to the PAK3 promoter at (+52/+60) both *in vivo* and *in vitro*. **A:** **** Electrophoretic Mobility Shift Assay (EMSA) showing protein binding to the (+52/+60) PAK3 promoter region. Lane 1 shows the presence of a DNA/protein complex when the (+52/+60) PAK3 promoter radioactively-labelled oligomer was incubated with protein cell lysate of Rat1a-J4 cells treated with doxycycline. This DNA/protein complex could be competed for with unlabelled (+52/+60) wild-type (WT) oligomers (lane 2), but not with a mutated (+52/+60) sequence oligomer (lane 3). The second panel shows supershifted complexes using anti-cJun (lane 4), -JunB (lane 5) and –JunD (lane 6) antibodies. **B:** Chromatin Immunoprecipitation Assay (ChIP) showing cJun binding to the (+52/+60) PAK3 promoter region. Rat1a-J4 cells were treated with doxycycline for 48 hrs, after which DNA and protein complexes were cross-linked and pulled down with an anti-cJun antibody. RT-PCR amplification off the pulled-down chromatin showed cJun binding to the (+52/+60) PAK3 promoter region in the presence of doxycycline.

As PAK3 promoter deletion and mutation assays suggested that AP-1 regulation on the PAK3 promoter occurs in the (+52/+60) region, we used *in vitro* and *in vivo* binding assays to investigate whether AP-1 is directly regulating PAK3 expression through binding this region of the PAK3 promoter. An Electophoretic Mobility Shift Assay (EMSA) was performed using a radiolabeled double-stranded oligomer spanning the (+52/+60) region. This *in vitro* binding assay showed the presence of a DNA/protein complex when radioactively-labeled (+52/+60) oligomers of the PAK3 promoter were incubated with protein cell lysate of Rat1a-J4 cells treated with doxycycline (Fig. 3A, lane 1). The presence of this DNA/protein complex was competed for with the addition of excess unlabeled (+52/+60) oligomers (lane 2), where these added oligomers contained the wildtype (+52/+60) sequence. The addition of excess mutated unlabeled (+52/+60) oligomers did not outcompete the binding of the seen DNA/protein complex (lane 3). Supershift analysis with cJun (lane 4), JunB (lane 5) and JunD (lane 6) antibodies suggest that the protein component of the DNA/protein complex contains cJun and to a lesser extend JunD. JunB was not found to play a role in the complex with the (+52/+60) oligomer. *In vivo* binding of cJun to the (+52/+60) PAK3 promoter region was shown using Chromatin Immunoprecipitation (ChIP) Assays, where our results show pull-down of cJun, using a specific cJun antibody, in the presence of doxycycline-treated Rat1a-J4 cell extracts accompanies the presence of the (+52/+60) region of the PAK3 promoter (Fig. 3B). Together, these results show that AP-1 is a transcriptional activator of PAK3 expression through direct binding to the (+52/+60) PAK3 promoter.

### PAK3 Plays a Key Role in the Cell Morphology and Migration Associated with AP-1-induced Transformation

**Figure 4:**
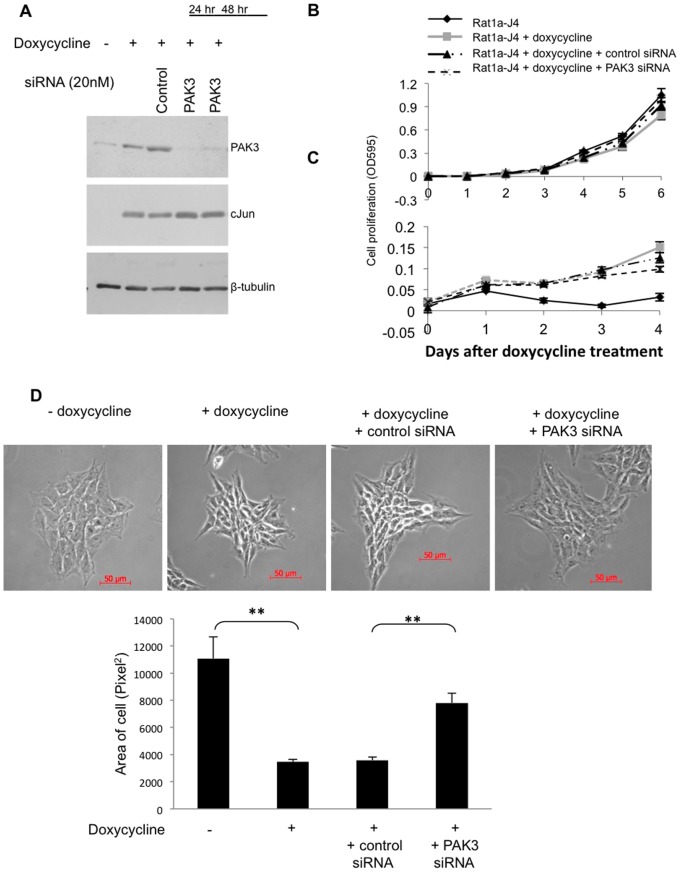
Inhibition of PAK3 in cJun/AP-1 over-expressing cells has no effect on proliferation, but results in a change in cell morphology. **A:** Western blot analysis confirming transient PAK3 inhibition using PAK3 siRNA in cJun expressing Rat1a-J4 cells. **B & C:** Cell proliferation assays (MTT) in doxycycline-induced cJun/AP-1 expressing cells with or without control-A or PAK3 siRNA in anchorage-dependent (**B**) and anchorage-independent (**C**) growth conditions. The results shown are the mean±S.E. of experiments performed in quadruplicate and repeated. **D:** Phase contrast microscopy of Rat1a-J4 cells grown in the absence and presence of doxycycline with the addition of either control or PAK3 siRNA. The bar graph shows the mean area (pixel^2^)±S.E. of forty cells of each condition over four fields of view quantitated with AxioVision 4.7 software. (*p≤0.05 and **p≤0.01).

Having established that PAK3 is an AP-1 responsive gene, the functional relevance of the increased levels of PAK3 within AP-1 transformed cells was investigated. To investigate whether elevated PAK3 is required for cJun/AP-1-induced cellular transformation, inhibition of PAK3 using siRNA was performed. Western blot analysis showed inhibition of PAK3 protein levels within 24 hours of transfection with PAK3 siRNA (Fig. 4A). As cJun/AP-1 induction associates with changes in proliferation, morphology and migration [1], these characteristics were investigated when PAK3 was inhibited in a cJun/AP-1 over-expressing cells. Cell proliferation assays in Rat1a-J4 cells, grown in the absence and presence of doxycycline to induce cJun expression, were performed for both anchorage-dependent (Fig. 4B) and anchorage-independent growth (Fig. 4C). Inhibition of PAK3 expression using PAK3 siRNA had no effect on cell proliferation. The growth advantage seen in cJun/AP-1 over-expressing cells grown in the anchorage-independent condition was also not altered when PAK3 expression was inhibited. While there was no change in proliferation when PAK3 was inhibited, a change in the cJun-induced cell morphology was observed. Phase contrast microscopy showed a distinctive change in the morphology of rat fibroblasts when cJun/AP-1 was over expressed to that of elongated, smaller spindle-shaped cells that pack more tightly together (Fig. 4D). When PAK3 was inhibited in cJun/AP-1 over-expressing cells, the morphology resembled that of control cells grown in the absence of doxycycline. Quantification of the changes in area occupied by these cells showed that PAK3 inhibition resulted in significantly larger cells compared to cJun/AP-1 over-expressing cells (Fig. 4D). This illustrates that PAK3 plays a role in the altered morphology associated with cJun/AP-1 transformation as inhibiting its expression caused a reversion in the cJun/AP-1 associated morphology.

**Figure 5:**
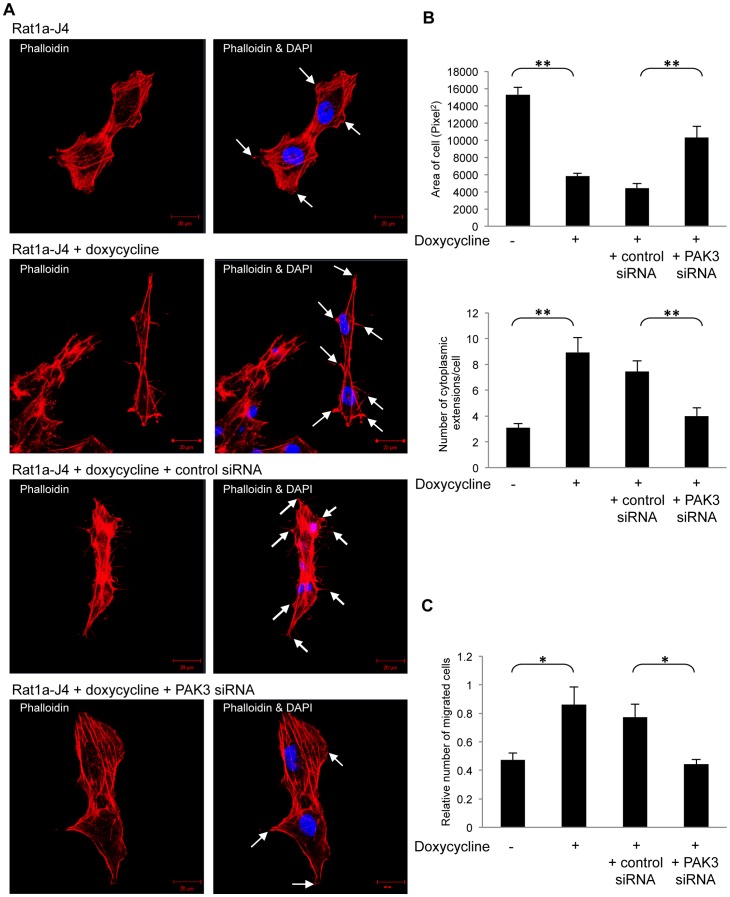
PAK3 inhibition affects actin organization and migration associated with cJun/AP-1 transformation. **A:** Fluorescent staining of polymeric F-actin in Rat1a-J4 cells grown in the absence of doxycycline (Rat1a-J4), in the presence of doxycycline (Rat1a-J4+doxycycline), in the presence of doxycycline with control siRNA (Rat1a-J4+doxycycline +control siRNA) and in the presence of doxycycline+PAK3 siRNA (Rat1a-J4+doxycycline +PAK3 siRNA) using phalloidin showed cytoskeletal rearrangements. DAPI stain was used to visualize the cell nuclei. Arrows point to cytoplasmic extensions. **B:** Quantitation of the changes in cell area (pixel^2^) (top panel) and number of cytoplasmic extensions (bottom panel) per cell from the captured fluorescent images. Cell area was calculated using AxioVision 4.7 Software. The results show the mean±S.E. over six fields of view. (**p≤0.01) **C:** Migration assays, using transwell chambers, with doxycycline-induced cJun/AP-1 over-expressing cells with either control-A or PAK3 siRNA. The results show the mean±S.E. of experiments performed in triplicate and repeated. (*p, 0.05).

Changes in cell morphology are often caused by cytoskeletal rearrangement and PAK3 has been shown to be essential for the organization of F-actin at the leading edge of submarginal cells [27]. We investigated whether the morphological changes associated with PAK3 inhibition were linked to changes in actin re-distribution. Actin staining assays were performed using phalloidin, which binds polymeric F-actin, to investigate actin reorganization in Rat1a-J4. Actin staining in cJun/AP-1 over-expressing Rat1a-J4 cells revealed an increased emergence of multiple actin-rich protrusions at the edges of the elongated, spindle-shaped cells, compared to control Rat1a-J4 cells (Fig. 5A). These actin-rich cellular extensions, along with the smaller spindle-like cellular morphology, were also seen in cJun/AP-1 transformed Rat1a-J4 cells transfected with control siRNA. When PAK3 was inhibited using siRNA in cJun/AP-1 expressing cells, there was a change in cell shape and a reduction in cellular protrusions. Quantification of the change in cell area occupied and the number of cytoplasmic extensions are shown in Fig. 5B. These results are in line with those obtained using phase contrast microscopy. These results indicate that PAK3 plays a role in the actin reorganization associated with cJun/AP-1 transformation.

Remodelling of the actin skeleton is known to produce the inner motive force for the migration of cells. Thus following the changes seen in actin reorganization of cJun/AP-1 over-expressing Rat1a-J4 cells and the effect PAK3 inhibition had on these changes, motility assays were performed. Transwell migration assays showed that PAK3 inhibition reduced the migration of cJun/AP-1 over-expressing cells (Fig. 5C). Together, these results show PAK3 is required for cyctoskeletal organization and the migratory potential of cJun/AP-1 transformed cells.

### PAK3 is Necessary for the Morphology and Migration of Transformed Human Fibroblasts

**Figure 6:**
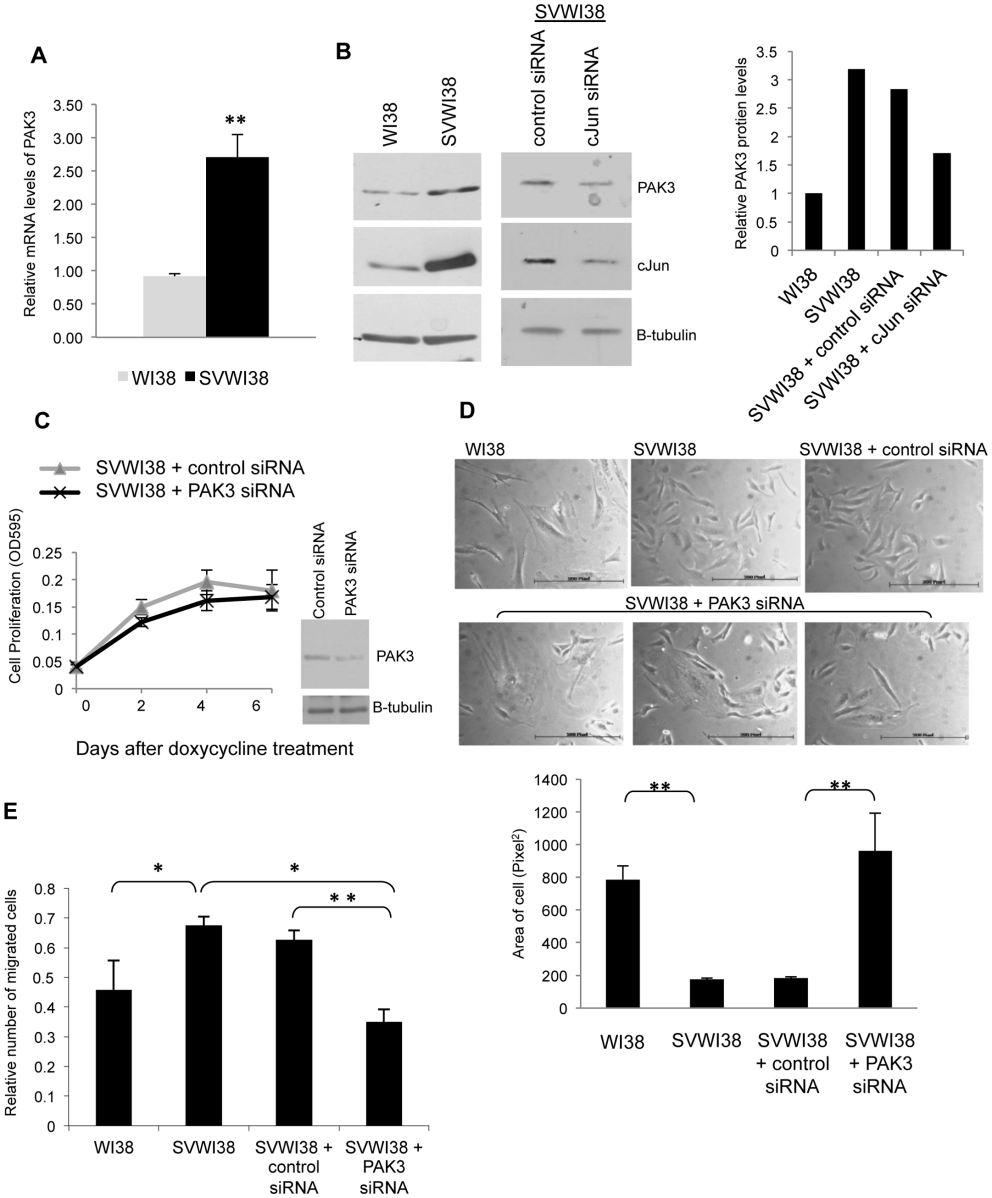
PAK3 expression is elevated in transformed SVWI38 fibroblasts and plays a role in their cell morphology and migration. **A:** Real time RT-PCR for PAK3 mRNA expression levels in normal, WI38, and transformed lung fibroblasts, SVWI38. The results show the mean±S.E. of at three independent experiments. (*p≤0.05 and **p≤0.01) **B:** Western blot analysis showing PAK3 and cJun expression in normal, WI38, and transformed lung fibroblasts, SVWI38, as well as in SVWI38 cells with transient knock-down of cJun using siRNA. The bar graph represents the quantitated levels of PAK3 protein relative to β-tubulin. **C:** Cell proliferation assays for SVWI38 cells transfected with control-A or PAK3 siRNA. The results show the mean±S.E. of experiments performed in quadruplicate. **Inset:** Western blot analysis shows the transient PAK3 knock-down using PAK3 siRNA in SVWI38 fibroblasts assayed. **D:** Phase contrast microscopy showing the morphology of WI38 fibroblasts compared to SVWI38 fibroblasts transfected with control-A or PAK3 siRNA after 48 hours. The bar graph shows the mean area (pixel^2^)±S.E. of sixty cells of each condition over six fields of view quantitated with AxioVision 4.7 software. (**p≤0.01). **E:** Transwell motility assays measuring the number of migrating cells of WI38 compared to SVWI38 cells transfected with control-A or PAK3 siRNA. The results show the mean ­± S.E. of the mean of experiments performed in triplicate and repeated. (**p≤0.01).

Having shown that elevated PAK3 expression in response to cJun/AP-1 over-expression associates with some of the phenotypes of AP-1 transformation in a rat fibroblast model system, we investigated a possible link of PAK3 and cJun in normal human fibroblast, WI38, and its transformed counterpart, SVWI38 cells. PAK3 mRNA levels were found to be significantly increased in the transformed fibroblast cells, SVWI38s, compared to the normal WI38s (Fig. 6A). Western blot analysis similarly showed increased PAK3 protein levels in the SVWI38 cells, compared to the WI38 cells (Fig. 6B). Elevated levels of PAK3 in SVWI38 cells associate with elevated cJun protein levels. These increased levels of cJun appear necessary to maintain the increased level of PAK3 as cJun knock-down in SVWI38 cells reduced the level of PAK3 protein. PAK3 inhibition in SVWI38 transformed cells had no effect on cell proliferation (Fig. 6C). Phase microscopy of cell morphology, showed that inhibition of PAK3 expression in SVWI38 cells resulted in flattened, enlarged and elongated cells, resembling the morphology of WI38 fibroblasts (Fig. 6D). Quantification of these results showed a significant increase in cell area in SVWI38 cells when PAK3 is inhibited. Motility assays showed that the inhibition of PAK3, using siRNA, in SVWI38 cells interfered with cell migration, reducing the migration of these cells to levels of that seen in normal WI38 cells (Fig. 6E). These results show a distinctive role for PAK3 in the cellular transformation, specifically within the morphological and migratory phenotype that is associated with elevated cJun/AP-1 expression.

## Discussion

Despite the deregulation of AP-1 being known to play a pivotal role in transformation of cells and the promotion and progression of tumourigenesis [28], very little is known about the mechanism with which it controls its diverse down-stream functions. With such varied targets, it is hypothesized that there is a group of AP-1 target genes responsible for inducing the transformed phenotype, as opposed to a single effector [12]. Differential gene expression analysis between control and cJun/AP-1 transformed cells identified PAK3 as a potential AP-1 target gene upregulated in response to cJun over-expression [16]. In this study, we report a relationship between AP-1 and PAK3 and show, in a rat model system, that over-expression of cJun results in elevated PAK3 mRNA and protein levels. In addition, we show that the PAK3 promoter is responsive to AP-1 via an interaction with the (+52/+60) PAK3 promoter region. This study is a first to show that cJun/AP-1 regulates the expression of a member of the Group 1 p21-Activated Kinases.

Activated cJun is highly present in many carcinomas [29]; [30] and its activity is needed for Ras, a Rho GTPase, -mediated morphological transformation [31]. PAK3, through its dynamic interaction with Rho GTPases [24] is known to be involved in cell morphology [17]. Our study showed that inhibition of PAK3 in AP-1 transformed cells repressed the transformed morphology associated with cJun/AP-1 over-expression and maintained a morphology more closely resembling control cells, outlining a role for PAK3 in the morphology of AP-1 transformed cells.

Although the Rho family of GTPases are thought to activate the family of PAK proteins, it is specifically the Rho GTPases CDC42 and Rac that are the main activators of PAKs [18]. CDC42 is thought to activate fillopodia formation, while the three isoforms of Rac (1, 2, 3) regulate actin polymerization at the periphery of the cell and control the development of membrane ruffles and lamellipodia [32]–[34]. Though the role of PAK proteins in these actin remodeling functions of CDC42 and Rac, PAK3, in particular, has been shown to be essential for the organization of F-actin at the leading edge of submarginal cells [27]. In this study we show a role for PAK3 in the altered morphology of AP-1 transformed cells and actin reorganization underlying these morphological changes. Our results suggest that PAK3 may be involved in the redistribution of F-actin to form the actin-rich protrusions on the tips of the smaller elongated AP-1 transformed cells. Our findings support work done by Amyere et al. (2000) [35] who show that when Rat1a fibroblasts are transformed with an oncogene, such as K-Ras, one major or many cellular protrusions emerge from the cells and these protrusions show active actin-rich ruffling at the tips. On further analysis of these F-actin tips, they showed that these ruffles were in fact curled lamelliopodia. Other studies have also shown that the activation of PAKs lead to the formation of lamelliopodia and motility of the cell [36]–[38]. This indicates that the results seen in this study implicate PAK3, specifically, in the cytoskeletal reorganization of F-actin in AP-1 transformed fibroblasts to result in the formation of lamelliopodia.

Remodeling of the actin in the cytoskeleton by regulatory proteins has been shown to play a distinctive role in cancer metastasis [39]. The Rho family of GTPases has also been shown to be vital regulators of cell movement in response to extracellular signals [40]; [41]. AP-1 activity is known to mediate motility and invasion [42], where knock-down of AP-1 reduces directional migration [31]. PAK1 and PAK2, the other members of the Group 1 PAK family, are activated by cellular cues that stimulate migration [43] with PAK1 directly initiating this movement [24]. Our results show that PAK3 inhibition in cJun/AP-1 over-expressing cells decreases the migratory potential of these cells, suggesting PAK3 may play a pivotal role in AP-1-associated migration.

While the inhibition of PAK3 had a significant effect on morphology and migration, this study showed it had no effect on the proliferative advantage see in transformed fibroblasts and compared to their normal counterparts (specifically within anchorage-independent growth conditions). Although this appears to suggest no role for PAK3 in proliferation, there is evidence to support that PAK1, PAK2 and PAK3 can play a compensatory role in proliferation after a loss of one of these proteins [44]. Further inhibition of PAK1 and PAK2 proteins alongside PAK3 inhibition would give a clearer understanding of the role PAK3 plays in the proliferation of AP-1 transformed cells. While PAK1, 2 and 3 may play a compensatory role in proliferation, these proteins are not generally thought to have overlapping functions. Interestingly, PAK3 like PAK1, has been showed to phosphorylate Raf1 and promote anchorage-independent growth [27]. Raf has been shown to cause transformation of polarized MDCK cells through the activation of Rac1 [45].

In summary, our results show PAK3 is an AP-1 regulated gene involved in maintaining the morphological and migratory phenotype as well as a functional role for PAK3 in AP-1 associated cellular transformation.

## Materials and Methods

### Cell Culture and Growth Conditions

Cells were grown in Dulbecco’s Modified Eagle’s Medium (DMEM) with 10% heat inactivated Fetal Calf Serum (FCS) (Gibco), 100 U/ml penicillin and 100 µg/ml streptomycin. Cells were grown at 37°C in a 5% CO_2_ incubator and passaged using 0.05% trypsin and EDTA. Rat1a-J4 cells, containing a doxycycline-inducible cJun construct, and Rat1a-GFP cells, containing a doxycycline-inducible green fluorescent protein (GFP) construct, were maintained by selection with 5 µg/ml blastacidin or induced for gene expression with 2 µg/ml doxycycline. Anchorage-dependant growth conditions allowed cells to adhere directly to the cell culture dish, while anchorage-independent conditions were mimicked by plating cells onto Poly-HEME (Sigma) coated culture dishes.

### Real-time RT-PCR and Primers

RNA was extracted using 80% Trizol (Invitrogen), according to the manufactures protocol. Complementary DNA (cDNA) first strand synthesis was performed with ImProm-II Reverse Transcriptase (Promega) and real-time RT-PCR was performed using the StepOne Real-Time PCR System (Applied Biosystems) and SYBR Green I mix (KAPA Biosystems), according to the manufactures instructions. Primer sequences were as follows: Rat PAK3 (F 5′-TCACTCCTGAGCAAAGTAAACG-3′; R 5′-TCCCAGAGACCAGATATCAACTT-3′; 112 bp product), human PAK3 (F 5′-ATGACTCTACCCACGGCAAG-3′; R 5′-TACTCAGCACCAGCATCA-3′; 136 bp product), rat GAPDH (F 5′-ATGACTCTACCCACGGCAAG-3′; R 5′-TACTCAGCACCAGCATCA-3′; 136 bp product) and human Gus B (F 5′-CTCATTTGGAATTTTGCCGATT-3′; R 5′-CCAAGTGAAGATCCCCTTTTT A-3′; 81 bp product). Gene expression of PAK3 was standardized relative to an internal control; for rat expression, GAPDH, and for human, Gus B. All samples were performed in triplicate and the relative mRNA expression level of the gene of interest was calculated using the 2^−ΔΔCT^ method [46].

### Western Blot Analysis

Protein was extracted in RIPA and separated by 10% SDS-PAGE and transferred to a Hybond™-ECL™ nitrocellulose membrane (Amersham Life Sciences). The membrane was blocked in 5% low fat milk in Tris-buffered saline with 1 x Tween and then incubated with primary antibodies: anti-PAK3 (sc-48826 (Santa Cruz)); anti-PAK1 (sc-882 (Santa Cruz)); anti-PAK2 (sc-7117 (Santa Cruz)); anti-cJun (sc-1694 (Santa Cruz)) and anti-β-tubulin (sc-9104 (Santa Cruz)). Primary antibodies were detected with horseradish peroxidase-conjugated secondary antibodies: anti-rabbit (#172-1019 (BioRad)) and anti-goat (sc-2020 (Santa Cruz)) and LumiGLO chemiluminescent substrate system (KPL). ImageJ software was used to quantitate protein expression levels, and included bar graphs represent the expression of the each protein relative to the β-tubulin for that sample.

### Promoter Analysis

#### Plasmid preparation

The (-2436/+149) rat PAK3 promoter region (GenBank Accession Number: NC_005120) was amplified using primers: F 5′-TG**ACGCGT**AGAGAAGGAAGCCAAGAATC-3′ (Mlu1 site in bold) and R 5′-CGCTCGAGGTGTAAGACCCCAGACAGTT-3′ (Xho1 site is underlined). PCR using high fidelity Expand Plus DNA Polymerase (Roche) was performed on rat genomic DNA, the products were sub-cloned into the pGEM-T Easy vector (Promega) and excised for cloning into the luciferase reported vector, pGL_3_-Basic (Promega) using Mlu1 and Xho1 restriction enzymes. The promoter construct was verified by sequencing.

#### Generation of PAK3 promoter deletion constructs

Promoter deletion constructs were made using the included, cloned Mlu1 site and restriction enzyme sites within the cloned promoter (EcoRI for the -2364/+149 construct; PstI for the −684/+149 construct and NsiI for the −179/+149 construct). Once a region was excised out, the remaining linearized plasmid was gel purified and the sticky ends were blunted-ended using T4 DNA Polymerase (Promega) before the plasmid was closed by self-ligation.

#### Site-directed mutagenesis of the (+52/+60) putative cJun binding site

The (+52/+60) putative cJun binding site has the wildtype sequence of TGACGTCA. This site in the (−179/+149) PAK3 promoter construct was mutated to ACGCGTTT (where the underlined regions highlight the mutate bases) through site-directed mutagenesis using the following primers: F 5′-GGTACGGTGCAGAGCCCAGGacgCGTtaTAGCATAGAAGAGCTAGG-3′ and R 5′-CCTAGCTCTTCTATGCTAtaACGcgtCCTGGGCTCTGCACCGTACC-3′ where the mutated bases are in lowercase and the incorporated Mlu1 site is underlined. After PCR amplification, Dpn1 (Promega) was used to digest the template plasmid before the products were transformed into JM109 highly competent cells (Promega). The introduced novel Mlu1 restriction enzyme sites were used to confirm that inclusion of the mutation.

#### Luciferase promoter assays

Promoter constructs were transfected into rat fibroblasts using TransFectin Lipid Reagent (BioRad). pRL-TK (Promega) was used as a control for transfection efficiency. cJun/AP-1 expression was induced with 2 µg/ml doxycycline in Rat1a-J4 cells, and transfection with pCMV-cJun plasmid in Rat1a cells. cJun knock-down was performed using 20 nM cJun siRNA (sc-29223, Santa Cruz Biotechnology) with a 3∶1 ratio of TransFectin Lipid Reagent (BioRad). Control-A siRNA (sc-37007, Santa Cruz Biotechnology) was used as an siRNA control. Cell lysates were prepared after 48 hours using Passive Lysis Buffer (Promega) and luciferase activity measured using the Dual-Luciferase® Reporter Assay Kit (Promega) and measured on the Glomax 96 Microplate luminometer (Promega).

### Electrophoretic Mobility Shift Assay (EMSA)

γ -^32^P-labled oligomers (20-mers) containing the (+52/+60) PAK3 promoter region were incubated with crude nuclear protein, extracted from Rat1a-J4 cells treated with 2 µg/ml doxycycline for 48 hrs, as well as poly(dI/dC) and 5× Incubation Buffer (100 mM HEPES (pH 7.9), 250 mM KCl, 2.5 mM DTT, 1 mM EDTA, 5 mM MgCl_2_, 20% Ficoll 400) after a 20 min incubation on ice. Protein-oligomer mixtures were incubated for a further 30 min on ice and then separated on a non-denaturing 5% polyacrylamide gel at 150V for approximately 2 hrs at 4°C in 1×TBE. For supershift analysis, anti-cJun (sc-1694 (Santa Cruz)), anti-JunB (sc-73 (Santa Cruz)) and anti-JunD (sc-74 (Santa Curz) antibodies were used. For oligomer competition, unlabeled (cold) wildtype and mutant were used in 75× molar excess.

### Chromatin Immunoprecipitation Assay (ChIP)

Protein-DNA complexes from untreated and 48 hour doxycycline-induced Rat1a-J4 cells were cross-linked with 1% formaldehyde and 0.125 M glycine, pH 2.5. Cells were re-suspended in lysis buffer (1% SDS, 5 mM EDTA, 50 mM Tris-Cl, pH 8.1, 1 X Complete Protease Inhibitor (Roche)) and sonicated to sheer chromatin to between 400–100-bps in length. Lysates was then diluted with dilution buffer (1% Triton X-100, 2 mM EDTA, 150 mM NaCl, 20 mM Tris-Cl, pH 8.1, 1 X Complete Protease Inhibitor) and chromatin was either immunoprecipitated with or without anti-cJun antibody (sc-44 (Santa Cruz)) and treated with pre-blocked protein-A agarose beads (Merck). Bead-bound complexes were recovered by centrifugation and washed sequentially with TSE I (0.1% SDS, 1% Triton X-100, 2 mM EDTA, 20 mM Tris-Cl, pH 8.1, 150 mM NaCl), TSE II (0.1% SDS, 1% Triton X-100, 2 mM EDTA, 20 mM Tris-Cl, pH 8.1, 500 mM NaCl), Buffer III (0.25 M LiCl, 1% NP-40, 1% Sodium Deoxycholate, 1 mM EDTA, 10 mM Tris-Cl, pH 8.1) and TE, pH 7.4. Bound chromatin was released from beads using elution buffer (1% SDS, 0.1 M NaHCO_3_) and released from formaldehyde cross-links by heating at 65°C overnight. RT-PCR was performed on the purified chromatin using primers spanning the (+52/+60) PAK3 promoter site (FWD: 5′ AGATTGGTCCCCAGTAGCCC 3′ and REV: 5′ ACCCCAGACAGTTTGCGG 3′).

### PAK3 Transient Knock-down

Transient PAK3 knock-down was performed using 20 nM PAK3 siRNA (βPAK sc-36182, Santa Cruz Biotechnology) with a 3∶1 ratio of TransFectin Lipid Reagent (BioRad). Control-A siRNA (sc-37007, Santa Cruz Biotechnology) was used as an siRNA control.

### Cell Proliferation Assay (MTT Assay)

MTT (3-[4,5-dimethylthiazol-2-yl]-2,5 diphenyl tetrazolium bromide) assays were performed when transfected cells were seeded into 96-well plates in normal growth medium with or without doxycycline. For anchorage-independent growth, the 96-well plates were coated with Poly-HEME prior to plating. Cell growth was measured every 24 hours by MTT assay according to the manufactures instructions (Roche).

### Actin Staining, Microscopy and Cell Shape Analysis

Rat1a-J4 cells, plated onto coverslips in a 35 mm dish, were transfected and treated with or without doxycycline for 48 hours. Cells were fixed in 4% paraformaldehyde, washed twice in 0.04% PBS-Tween and blocked in 1% BSA. Actin was labeled with 50 µg/ml Phalloidin-Tetramethylrodamine B isothiocyanate (Sigma) in 15% BSA for 30 min at room temperature. DAPI was used to stain the cell nuclei. Phase pictures were taken using a standard fluorescence microscope, while the phalloidin images were viewed using the Zeiss LSM 510 Meta fluorescent microscope and captured using the ZEN 2009 camera with associated AxioVision software (version 4.7). Human fibroblasts, WI38 and SVWI38 cells viewed under phase contrast microscopy were photographed live using the Motic MotiCam digital microscope. AxioVision 4.7 software was used measure changes in cell area over four or six fields of view.

### Motility Assays

12-well-plate Transwell migration chambers (Costar, Cambridge MA) with an 8 µm pores were placed into lower chambers containing 15% FCS-containing media, and cells were plated in 1% FCS-containing media in the top chamber. Migration through the membrane was allowed for 24 hours, after which the cells on top of the membrane were removed with a cotton swab and the cells attached to bottom of the membrane were fixed in methanol and stained with Crystal violet. Results were quantified using ImageJ software.

### Statistical Analysis

All experiments were performed in triplicate and were represented as the mean±standard error of the mean. A Student’s *t-*test was used to compare groups of samples and a p-value of less than 0.05 was considered statistically significant. P-values ≤0.05 were marked with a (*), while p-values ≤0.01 were marked with (**).
